# Resection of a huge mediastinal well-differentiated liposarcoma involving left thoracic cavity

**DOI:** 10.1186/s13019-019-0965-0

**Published:** 2019-08-06

**Authors:** Ming Zhang, Shaoqin Zhang, Hao Shi, Weidong Li, Zhengliang Wei

**Affiliations:** 1 Department of Cardiothoracic Surgery, Shengzhou Renmin Hospital, Shaoxing, 312400 China; 2 Department of Cardiothoracic Surgery, the First Affiliated Hospital, School of Medicine, Zhejiang University, Hangzhou, 310003 China

**Keywords:** Lipoma, Liposarcoma, Mediastinum tumor, Surgery

## Abstract

**Background:**

Mediastinal lipoma/liposarcoma is a rare tumor of the mediastinum.

**Case presentation:**

This article reported one case of giant anterior superior mediastinum well-differentiated liposarcoma involving the left thoracic cavity with symptom of dysphagia. The mediastinum liposarcoma was completely resected through a left thoracotomy. Histologic examination and molecular pathological test clarified the diagnosis as well-differentiated mediastinal liposarcoma. There has been no evidence of recurrence during the 8 months follow-up.

**Conclusion:**

Molecular pathological examination of the MDM2, CDK4 and p16 gene in tumors provides the diagnostic gold standard in distinguishing well-differentiated liposarcoma from lipoma. Complete surgical resection is the first-line treatment choice for mediastinal lipoma/ liposarcoma.

## Introduction

Lipoma and well-differentiated liposarcoma are well-circumscribed mesenchymal tumor which originate from adipose tissue [1]. Mediastinal lipoma and liposarcoma are rare tumors of the mediastinum, which mainly occurred in the anterior mediastinum, and constituted 1.6–2.5% of primary mediastinal tumors [2]. In this article, we reported one case of anterior superior mediastinum well-differentiated liposarcoma involving the left thoracic cavity with symptom of dysphagia.

## Case report

**Fig. 1:**
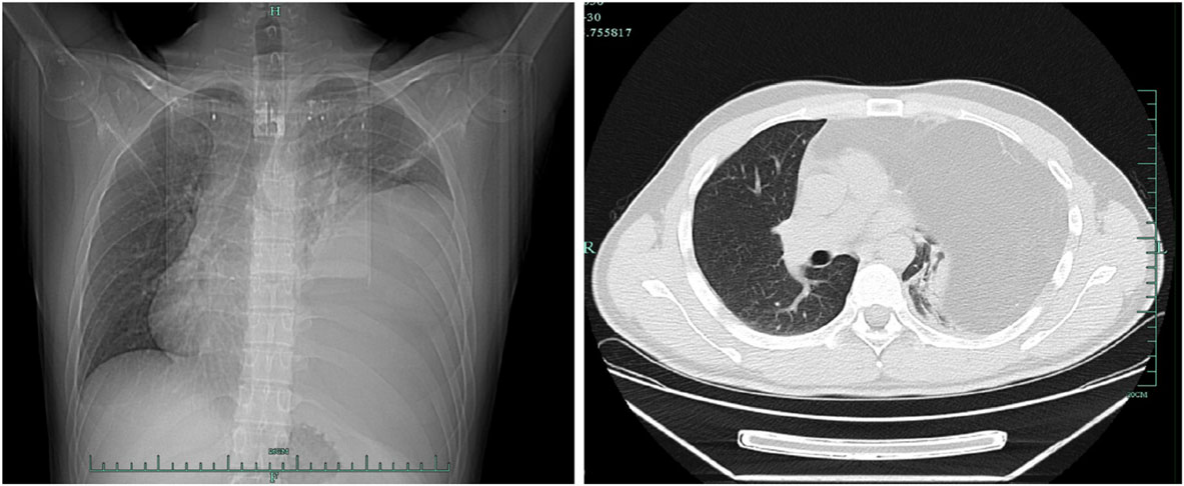
Chest CT scan revealed a huge mass with fat density in the anterior superior mediastinum and left lung field, the inferior lobe of left lung was compressed, trachea and heart shifted to the right side

**Fig. 2:**
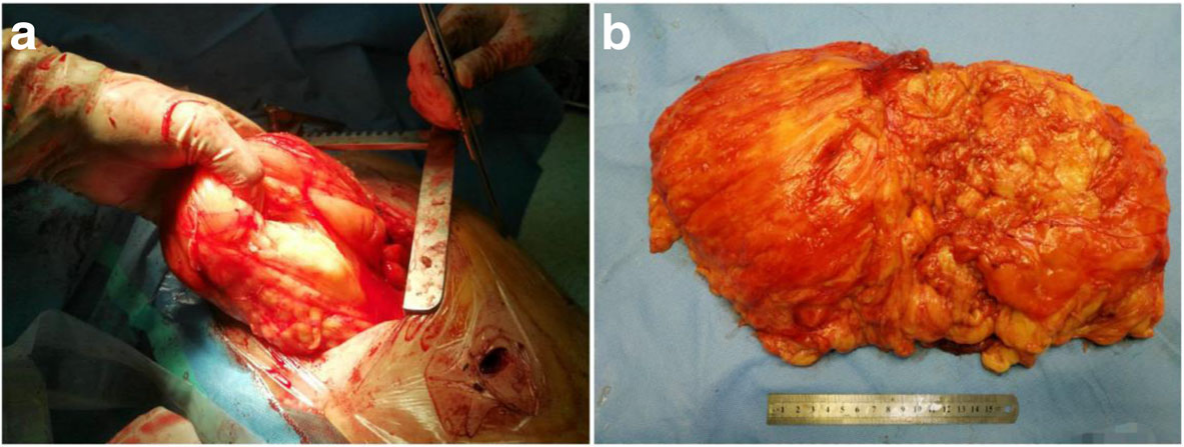
Gross morphology of the mediastinal lipoma during the operation (**a**) and after complete resection (**b**)

**Fig. 3:**
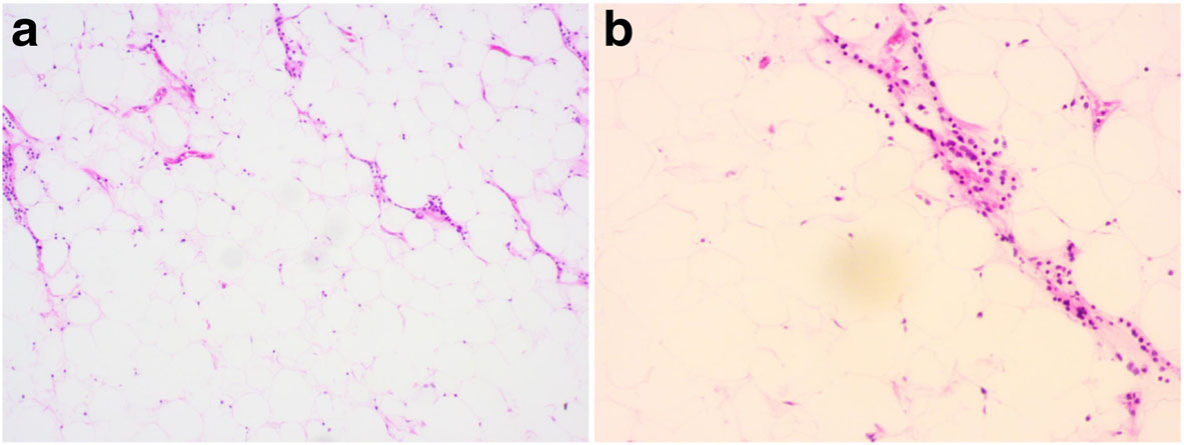
Histologic examination revealed that the tumor was formed by well differentiated adipose tissue (**a**), and several degenerative cells could be found among the adipose tissue (**b**)

**Fig. 4:**
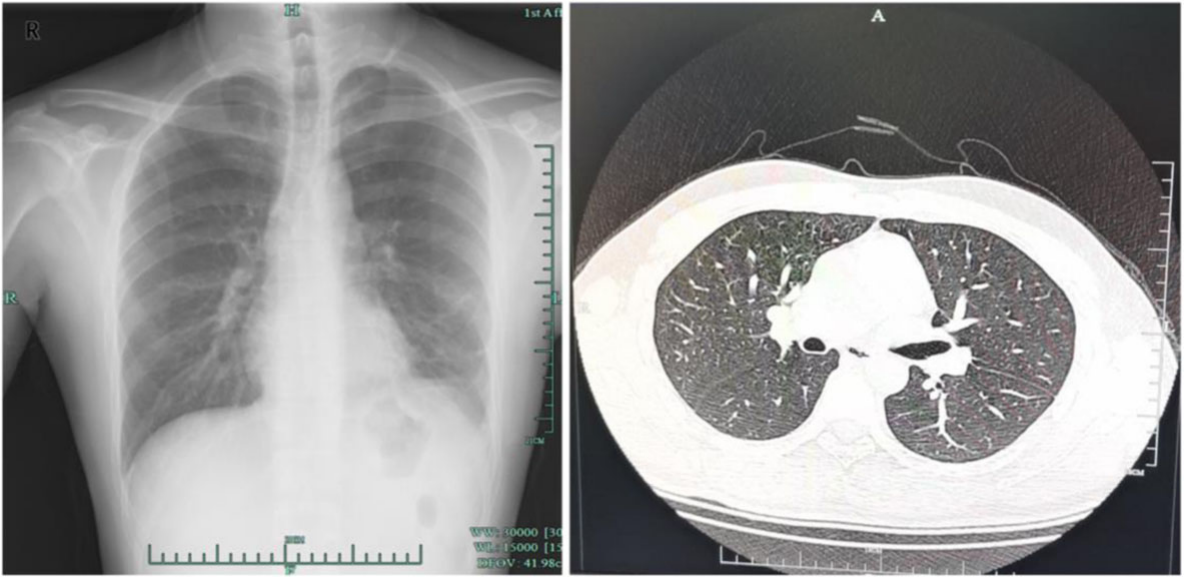
Chest X ray and CT scan of the patient 8 months after the surgery, which showed no evidence of recurrence

A 30-year-old man presented with dysphagia over 3 months was admitted to our hospital. Chest CT scan revealed a huge mass with fat density in the anterior superior mediastinum and left lung field, the inferior lobe of left lung was compressed, trachea and heart shifted to the right side (Fig. 1). Echocardiography showed the heart shifting towards lower right side. And the cardiac function was normal (EF: 71%). All tumor markers, except ferritin with a high value of 420.9 ng/mL, showed normal values. A left thoracotomy was performed through the 3th intercostal. Intraoperatively, a 20 × 30 cm mass was found firmly attached in the anterior superior mediastinum, and invaded into the left thoracic cavity, which occupied 2/3 space of the left thoracic cavity and oppressed the left lung (Fig. 2a). The boundary of the tumor was clear, which had some adhesion to the chest wall, and the tumor was soft and rich in blood supply (Fig. 2b). Histologic examination revealed that the tumor was formed by well differentiated adipose tissue, and several degenerative cells could be found among the adipose tissue (Fig. 3). Fluorescence In suit Hybridization (FISH) test confirmed that the tumor was MDM2 gene positive, which clarified the diagnosis as well-differentiated mediastinal liposarcoma. The patient received routine treatment and nursing postoperatively. The chest drainage was removed on the 6th day after the surgery, and the patient was discharged on the 7th day after the surgery. The patient kept regular follow-up with chest X ray and CT scan. There has been no evidence of recurrence during the 8 months follow-up (Fig. 4).

## Discussion

Mediastinal lipoma and well-differentiated liposarcoma usually grow slowly and remain asymptomatic. When these tumors reach a huge size, which compress the adjacent structures, they will cause symptoms such as superior vena cava syndrome, Horner’s syndrome, dysphagia, dyspnea, cough, spinal nerve paralysis, tachycardia, and heart failure [3–6].

There are five histological types of liposarcomas: well-differentiated, mucus, dedifferentiated, pleomorphic, and round cell. Well-differentiated liposarcoma is the most common type of liposarcoma, which can be further classified into three types: lipoma-like, sclerosis, and inflammatory [7]. Sometimes it is difficult to differentiate lipoma and lipoma-like well-differentiated liposarcoma, especially when the tumor is giant in size. On CT scan, both of these two tumors have homogeneous fat attenuation of approximately -100HU [1], while the fibrous septa of well-differentiated liposarcoma may be thicker, more irregular, or more nodular than lipoma [8]. MRI is more helpful to determine the soft tissue involvement, which has an 83% accuracy rate on diagnosing well-circumscribed liposarcomas [9]. Molecular pathological examination of the MDM2, CDK4 and p16 gene in tumors provides the diagnostic gold standard in distinguishing well-differentiated liposarcoma from lipoma [10]. MDM2 can inhibit the transcriptional activity of p53, which inhibits the tumor suppressor function of p53. CDK4 can promote the progression of cell cycle from G1 phase to synthesis phase, which accelerates cell proliferation. p16 can decelerate the cell cycle progression from G1 phase to synthesis phase, which acts as a tumor suppressor. Using the combination of MDM2, CDK4 and p16 is helpful in distinguishing well-differentiated liposarcoma from lipoma, and it had been suggested that the use of fluorescence in situ hybridization (FISH) to access MDM2 gene amplification is more sensitive and specific than immunohistochemistry in distinguishing well-differentiated liposarcoma from lipoma [10].

Neither lipoma nor liposarcoma is sensitive to chemotherapy or radiotherapy, and complete surgical resection is the first-line treatment choice [5]. When the tumor is enormous and solid, it is inadequate to use the thoracoscopic approach since complete tumor resection maybe difficult using this approach. Thus, extensive thoracotomy or standard median sternotomy is needed for this situation to provide a better exposure. In some cases when a large tumor compressing the heart, extracorporeal circulation can be used to ensure the safety and reliability [4].

In this case, the essential of the operation was to expose the tumor clearly and to resect it completely. In order to achieve those purposes, we chose extensive thoracotomy through the 3th intercostal instead of thoracoscopic approach to provide a better exposure and to ensure the safety of the surgery. The technical key point of excising such a huge mass is to find the pedicle of the tumor in the first place, and then isolate the tumor along the pedicle.

## Conclusion

Mediastinal lipoma and well-differentiated liposarcoma are well-circumscribed mesenchymal tumors of the mediastinum, which can cause various symptoms due to the tumor mass effect. Chest CT scan and MRI can help clarify the diagnosis, and molecular pathological examination of the MDM2, CDK4 and p16 gene in tumors provides the diagnostic gold standard in distinguishing well-differentiated liposarcoma from lipoma. Complete surgical resection is the first-line treatment choice for mediastinal lipoma/ liposarcoma.

## Data Availability

Please contact author for data requests.

## Abbreviations

FISH: Fluorescence In suit Hybridization
